# Clinical Feasibility of Reduced Field-of-View Diffusion-Weighted Magnetic Resonance Imaging with Computed Diffusion-Weighted Imaging Technique in Breast Cancer Patients

**DOI:** 10.3390/diagnostics10080538

**Published:** 2020-07-30

**Authors:** Eun Cho, Jin Hwa Lee, Hye Jin Baek, Ji Young Ha, Kyeong Hwa Ryu, Sung Eun Park, Jin Il Moon, Sung-Min Gho, Tetsuya Wakayama

**Affiliations:** 1 Department of Radiology, Gyeongsang National University School of Medicine, Gyeongsang National University Changwon Hospital, Changwon 51472, Korea; sgeisilver@gmail.com (E.C.); sartre81@gmail.com (H.J.B.); wonpiece@gmail.com (J.Y.H.); ryukh0329@gmail.com (K.H.R.); uneyes@hanmail.com (S.E.P.); drlotus@naver.com (J.I.M.); 2 Department of Radiology, Dong-A University College of Medicine, Busan 49201, Korea; 3 MR collaboration and Development, GE Healthcare, Seoul 04637, Korea; sungmin.gho@ge.com; 4 MR collaboration and Development, GE Healthcare, Tokyo 191-0065, Japan; TetsuyaWakayama@ge.com

**Keywords:** reduced field-of-view, diffusion-weighted imaging, computed diffusion-weighted imaging, breast cancer

## Abstract

Background: We evaluated the feasibility of the reduced field-of-view (rFOV) diffusion-weighted imaging (DWI) with computed DWI technique by comparison and analysis of the inter-method agreement among acquired rFOV DWI (rFOVA), rFOV DWI with computed DWI technique (rFOVS), and dynamic contrast-enhanced (DCE) magnetic resonance imaging (MRI) in patients with breast cancer. Methods: A total of 130 patients with biopsy-proven breast cancers who underwent breast MRI from April 2017 to December 2017 were included in this study. The rFOVS were reformatted by calculation of the apparent diffusion coefficient curve obtained from rFOVA *b* = 0 s/mm^2^ and *b* = 500 s/mm^2^. Visual assessment of the image quality of rFOVA *b* = 1000 s/mm^2^, rFOVS, and DCE MRI was performed using a four-point grading system. Morphologic analyses of the index cancer was performed on rFOVA, rFOVS, and DCE MRI. The signal-to-noise ratio (SNR), contrast-to-noise ratio (CNR), and contrast of tumor-to-parenchyma (TPC) were calculated. Results: Image quality scores with rFOVA, rFOVS, and DCE MRI were not significantly different (*p* = 0.357). Lesion analysis of shape, margin, and size of the index cancer also did not show significant differences among the three sequences (*p* = 0.858, *p* = 0.242, and *p* = 0.858, respectively). SNR, CNR, and TPC of DCE MRI were significantly higher than those of rFOVA and rFOVS (*p* < 0.001, *p* = 0.001, and *p* = 0.016, respectively). Significant differences were not found between the SNR, CNR, and TPC of rFOVA and those of rFOVS (*p* > 0.999, *p* > 0.999, and *p* > 0.999, respectively). Conclusion: The rFOVA and rFOVS showed nearly equivalent levels of image quality required for morphological analysis of the tumors and for lesion conspicuity compared with DCE MRI.

## 1. Introduction

In recent decades, breast magnetic resonance imaging (MRI) has been widely used in oncology patients because of its high sensitivity [1,2,3]. Due to the difference in enhancement levels between normal parenchyma and malignant tissue, dynamic contrast-enhanced (DCE) MRI plays an important role in everyday practice in the detection and diagnosis of breast cancer [4,5]. Moreover, by applying novel techniques to breast MRI, image quality and diagnostic value are being improved [6,7,8]. Compared to other traditional or emerging modalities, such as mammography, digital breast tomosynthesis (DBT), contrast-enhanced digital mammography (CEDM) or digital breast tomosynthesis (CEDBT), cone beam breast computed tomography (CBBCT), and contrast-enhanced dedicated breast computed tomography (CEDBCT), breast MRI offers the highest cancer detection rate and also has the advantage of avoiding radiation hazard. However, DCE MRI requires intravenous injection of a gadolinium-based contrast agent, which could have potential adverse effects, such as retention of the contrast agent in the tissues and allergic reactions [9,10,11]. Moreover, DCE MRI is also associated with increased imaging acquisition times and cost. For these reasons, several methods or protocols have been proposed without the use of intravenous contrast agent for detecting and characterizing the breast lesions [12,13,14].

Diffusion-weighted imaging (DWI) is a widely used functional MRI technique and one of the most promising noninvasive techniques that does not require administration of a contrast agent [15,16]. DWI can reflect the cellularity of the lesion by measuring the movement of water molecules in the tissue [17]. Malignant lesions have high cellularity and restricted diffusibility of water molecules in the tissue; thus, DWI can help discriminate a malignant lesion from a benign one [18,19].

**Figure 1:**
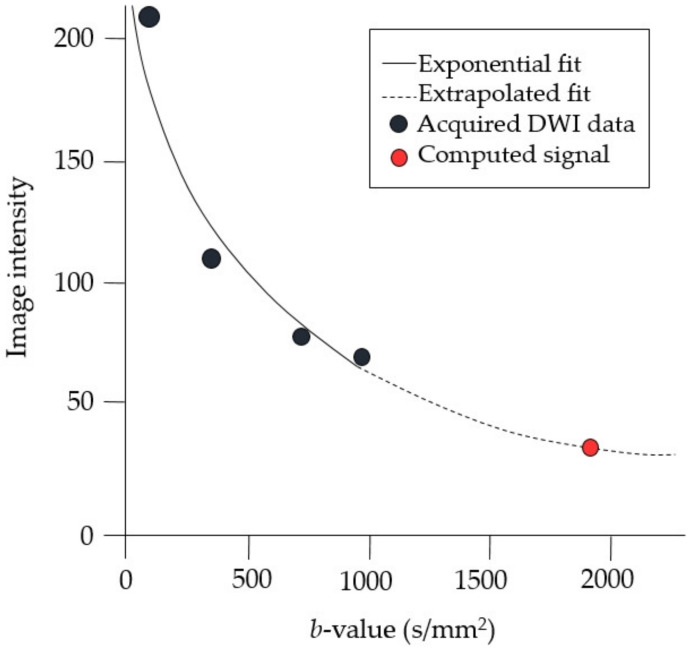
Principles of computed diffusion-weighted imaging (DWI) technique. The technique is based on the principle of DWI, wherein the values of apparent diffusion coefficient of various *b*-values were fit to an exponential curve.

Computed DWI is a computation technique that uses DWI acquired with at least two different lower *b*-values to obtain a new DWI with a higher *b*-value, using calculation on a voxel-by-voxel basis (Figure 1).

The technique is based on the principle of DWI in which the values of the apparent diffusion coefficient (ADC) of various *b*-values are fit into an exponential curve represented by the following equation [20,21,22]:

(1) $$S\left(b\right)=S\left(0\right)e^{-b\cdot ADC}$$

where $S\left(0\right)$ is the signal intensity at a *b* = 0 s/mm^2^. Once the value of ADC is known, it can be used to extrapolate the expected signal intensity for each imaging voxel to any computed *b-*value, *b_c_*, as follows:

(2) $$S\left(b_{c}\right)=S^{*}\left(0\right)e^{-b_{c}\cdot ADC^{*}}$$

where $S^{*}\left(0\right)$ and $ADC^{*}$ are the per-voxel estimates of $S\left(0\right)$ and ADC, respectively. Thus, computed diffusion-weighted images can be obtained. High *b-*value images obtained by the computed DWI technique have been shown to have high sensitivity and improved image quality that can help in detection of breast cancer [23,24,25,26,27].

DWI with reduced field-of-view (rFOV) technique can provide images with high spatial resolution by using a two-dimensional radiofrequency pulse that excites small areas and reduces artifacts. This technique has been applied mainly for imaging the spine, brain, pancreas, and prostate [28,29,30,31], but to our knowledge, there are very few studies with regard to breast imaging [32,33,34,35].

We focused on the merits of combining the advantages of the above two DWI MRI techniques in the breast cancer patient. We hypothesized that a high resolution of various *b-*value images could be obtained by applying the above two techniques while reducing imaging acquisition time. Therefore, the purpose of this study was to evaluate the clinical feasibility of rFOV DWI with computed DWI technique in patients with breast cancer by performing a comparison and analysis of the inter-method agreement among the acquired rFOV DWI (rFOVA), rFOV DWI with computed DWI technique (synthetic rFOV DWI; rFOVS), and DCE MRI.

## 2. Materials and Methods

This study was of a retrospective design and was approved by the Institutional Review Board of our institution (DAUHIRB 18-128, approval date: 30 Jun 2018). The requirement for informed consent was waived.

### 2.1. Subjects

We indentified 154 women who underwent preoperative breast MRI evaluation from April 2017 to December 2017. Of those women, only patients with primary biopsy-proven malignant breast tumors were included. We excluded patients with rFOV DWI unsuitable for tumor evaluation (*n* = 15), who had undergone neoadjuvant chemotherapy (*n* = 7), and who had undergone vacuum-assisted biopsy (*n* = 2). In total, 130 women with biopsy-proven malignant breast tumors were included.

### 2.2. MRI Acquisition

Imaging was performed on a 3.0 Tesla (T) whole-body MRI scanner (GE Discovery MR750, GE Healthcare, Waukesha, WI, USA) using a 8-channel phased-array breast coil. Bilateral breast imaging was performed using the following protocol: an axial T2-weighted sequence with fat suppression (repetition time/echo time (TR/TE), 8084/106 ms; flip angle, 111°; 3.0-mm thickness without an interslice gap; FOV, 320 × 320 mm^2^; matrix size, 320 × 256; number of excitations (NEX), 3.0; acquisition time, 261 s), 3D T1-weighted volume imaging for breast assessment (VIBRANT) dynamic gradient-echo sequence with intravenous bolus injection of 0.1 mmol/kg gadoterate meglumine (Dotarem, Guerbet) (TR/TE, 4.1/1.7 ms; flip angle, 10°; 1.0 mm thickness without an interslice gap; FOV, 320 × 320 mm^2^; matrix size, 320 × 320; NEX, 0.7; one unenhanced and five contrast-enhanced acquisitions; acquisition time, 374 s), and single-shot echo-planar-imaging (SS-EPI) DWI (TR/TE, 5002/55 ms; flip angle, 90°; 4.0 mm thickness without an interslice gap; FOV, 320 × 320 mm^2^; matrix size, 128 × 128; NEX, 6.0; *b-*value, 1000 s/mm^2^; acquisition time, 270 s).

The target for the rFOV DWI (FOCUS DWI; GE Healthcare) was determined by examining noncontrast-enhanced T2-weighted images with fat suppression and SS-EPI DWI through considering the findings on mammography and ultrasonography, which was performed before MRI acqusition by the radiologist. Bilateral shimming was performed by placing small rectangular shim boxes where the target lesion was deemed to be present. Diffusion-weighted gradients were applied in three orthogonal directions by use of two *b-*values, 500 s/mm^2^ and 1000 s/mm^2^. The scanning parameters of rFOVA were as follows: *b-*value 500 s/mm^2^ rFOV DWI (TR/TE, 3000/52 ms; flip angle, 90°; 4.0 mm thickness without an interslice gap; FOV, 100 × 50 mm^2^; matrix size, 128 × 64; NEX, 12.0; *b-*value, 500 s/mm^2^; acquisition time, 113 s) and *b-*value 1000 s/mm^2^ rFOV DWI (TR/TE, 3000/58 ms; flip angle, 90°; 4.0 mm thickness without an interslice gap; FOV, 100 × 50 mm^2^; matrix size, 128 × 64; NEX, 16.0; *b-*value, 1000 s/mm^2^; acquisition time, 153 s). Image acquisition parameters for rFOV DWI and DCE MRI are summarized in Table 1.

The axial T2-weighted sequence with fat suppression, SS-EPI DWI, rFOV DWI, and DCE MRI series were obtained in this order.

ADC maps were calculated by using an exponential curve fit, incorporating the signal intensity at two *b-*values on each voxel location. The image sets of rFOV DWI *b-*values 0 s/mm^2^ and 500 s/mm^2^ were used to create image sets of rFOVS with *b-*values of 1000 (S-1000), 1500 (S-1500), and 2000 s/mm^2^ (S-2000). The rFOVS image sets were reconstructed using the commercially available software MAGiC DWI (GE Healthcare).

### 2.3. MR Image Analysis

#### 2.3.1. Qualitative Analysis

The image sets of S-1000, S-1500, and S-2000 of rFOVS; those of rFOVA *b* = 1000 s/mm^2^ (rFOVA-1000); and those of DCE MRI were reviewed by two radiologists, J.H.L with 17 years experience in breast imaging (radiologist 1) and E.C with 5 years experience in breast imaging (radiologist 2). The two readers reviewed the same MR images and a consensus was reached in cases of disagreement. Imaging evaluation was performed in separate sessions for each image set. There was an interval of 1 week between the sessions for each image set. Every image set was read in a random order, and both radiologists were blinded to the MRI sequences and the clinical information of the patients. The window levels were set to be equivalent for image analysis. For each image set, a four-point grading system was used for qualitative analysis of the image quality: 4―no problems were noticed in the image; 3―image suffered from only minor degradation and was suitable for evaluation; 2―image quality was not good, but could be used for evaluation; and 1―poor image quality precluded assessment of the target lesion.

We selected a *b*-value image set with the highest image quality score among the S-1000, S-1500, and S-2000 of rFOVS. The analysis was performed in the *b*-value image set with the best image quality among the S-1000, S-1500, and S-2000 of rFOVS selected. Morphological analysis was perfomed only for the index cancer. Masses were subjected to morphological analysis and size measurement of the index cancer, and non-mass enhancement lesions were excluded from the morphological analysis. The index cancer was analyzed according to the descriptors used in the fifth edition of the Breast Imaging and Reporting Data System (BI-RADS) [36]. The shape and margin descriptors of the MRI BI-RADS lexicon were used for analysis of the lesion on rFOVS, rFOVA, and DCE MRI. The shapes were classified as oval, round, or irregular, and the margins were classified as circumscribed, irregular, or spiculated. The size of the index tumor was also measured on the rFOVS, rFOVA, and DCE MRI. The *b*-value image set with the best image quality among the S-1000, S-1500, and S-2000 of rFOVS, rFOVA-1000 images, and subtraction images and nonsubtraction images of the second phase of DCE MRI were evaluated.

#### 2.3.2. Quantitative Analysis

A radiologist with 5 years experience (E.C., radiologist 2) in breast imaging calculated the signal-to-noise ratio (SNR), contrast-to-noise ratio (CNR), and tumor-to-parenchymal contrast (TPC) on a workstation (AW volumeshare 7, GE Healthcare, Waukesha, WI, USA). The tumor regions-of-interest (ROIs) were manually drawn to delineate the borders of the tumors. The ROI of the normal parenchyma was drawn on normal breast fibroglandular tissue without enhancement in the contralateral breast in the case of DCE MRI. As for rFOVS and rFOVA, the normal parenchymal ROI was drawn on the fibroglandular tissue as far away from the index cancer as possible. The same tumor ROI and normal parenchymal ROI were also used to measure the ADC values of rFOVS and rFOVA.

For measuring the background noise, three circular ROIs with a diameter of 2 cm were placed outside the body (right, front, and left side of the breast) on the slices that showed the maximal diameter of the tumor in the phase-encoding direction. The radiologist averaged the standard deviations (SD, $\sigma_{\mathrm{background}}$) of the three circular ROIs to calculate the SNR.

SNR, CNR, and TPC of rFOVS, rFOVA, and DCE MRI for each lesion were calculated using the following equations:

(3) $$\mathrm{SNR}\;=\frac{S_{\mathrm{tumor}}}{\sigma_{\mathrm{background}}},$$

(4) $$\mathrm{CNR}\;=\frac{\left|S_{\mathrm{tumor}}-{\;S}_{\mathrm{parenchyma}}\right|}{\sqrt{\sigma_{\mathrm{tumor}}{}^{2}+{\;\sigma }_{\mathrm{parenchyma}}{}^{2}}}.$$

(5) $$\mathrm{TPC}\;=\frac{S_{\mathrm{tumor}}}{S_{\mathrm{parenchyma}}},$$

where $S_{\mathrm{tumor}}$ is the signal intensity of the breast tumor, $S_{\mathrm{parenchyma}}$ the signal intensity of the normal breast parenchyma, $\sigma_{\mathrm{tumor}}$ the standard deviation of the tumor signal intensity, $\sigma_{\mathrm{parenchyma}}$ the standard deviation of the normal breast parenchymal signal intensity, and $\sigma_{\mathrm{background}}$ the standard deviation of the background signal.

### 2.4. Statistical Analysis

One-way analysis of variance (ANOVA) test was used to compare the image quality scores (ordinal variable). This test was also used to compare the quantitative parameters, namely, tumor size, SNR, CNR, and TPC (continuous variables). To compare the categorical variables, namely, qualitative parameters from the morphologic assessment of the index tumor, we used the chi-square test. The agreement between the tumor size measurement on rFOVS, rFOVA, and DCE MRI and the pathological tumor size was determined using boxplots, wherein the mean difference of the index cancer size was used.

All statistical analyses in this study were performed with SPSS version 23.0 software (IBM SPSS Statistics, Chicago, IL, USA). *p* < 0.05 was considered to have statistical significance.

## 3. Results

### 3.1. Patients

A total of 130 patients had 130 malignant tumors in the breast. The mean and standard deviations of the patient age at diagnosis were 52.7 ± 10.3 years (range of 34–87 years). The mean tumor size was 27.5 ± 20.5 mm (range of 3–108 mm).

Of the 130 tumors, 91 (70.0%) were invasive ductal carcinomas; 7 (5.4%) were invasive lobular carcinomas; and 10 (7.7%) were other invasive cancers including invasive tubular carcinoma, mucinous carcinoma, medullary carcinoma, micropapillary carcinoma, metaplastic carcinoma, and malignant phyllodes tumor. Furthermore, 22 tumors (16.9%) were ductal carcinoma in situ.

The size of the tumor ROI was set the same for rFOVA-1000, srFOVS, and DCE MRI; the average ROI size was 4173.5 ± 522.0 mm^2^.

The image acquisition time of rFOVA-1000 was 153 s, and that of rFOVA-500 was 113 s. Image sets of rFOVS were obtained from the rFOV DWI *b-*values, 0 s/mm^2^ and 500 s/mm^2^, without requiring additional imaging acquisition time. The image acquisition time with DCE MRI was 374 s.

### 3.2. Qualitative Analysis

**Figure 2:**
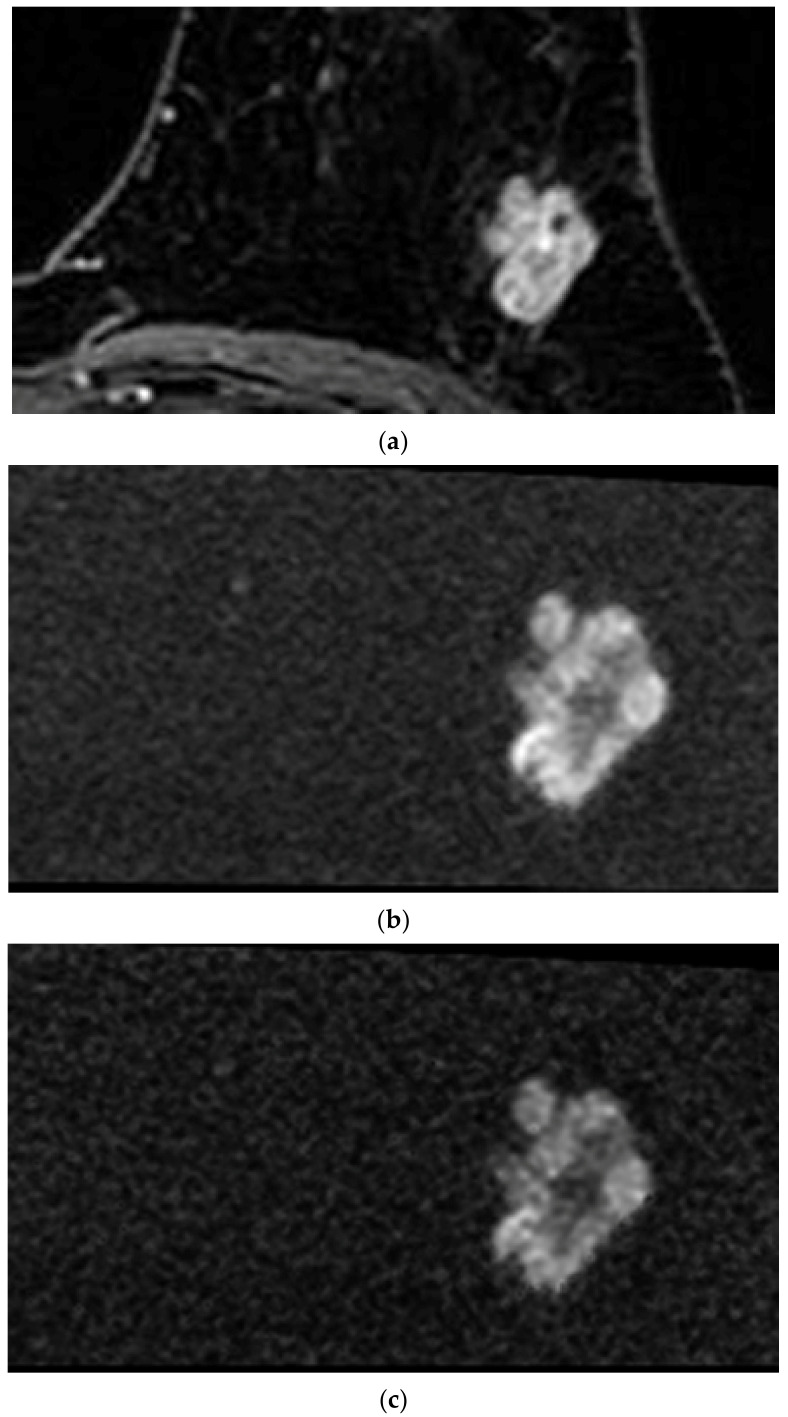
A 63-year old woman who had medullary carcinoma in her right breast. Axial contrast-enhanced T1-weighted fat-suppressed MRI (**a**) shows a 19 × 25 mm-sized enhancing mass with irregular shape, irregular margin, and heterogeneous and marked enhancement in the left 2 o’clock direction. rFOVA-1000 ^a^ (**b**) and rFOVS-1500 ^b^ (**c**) reveals the well-delineated index tumor, and the irregularity of the shape and the margin of the index tumor is also well depicted. ^a^ rFOVA-1000: acquired reduced field-of-view diffusion-weighted images with *b*-value 1000 s/mm^2^. ^b^ rFOVS-1500: synthetic reduced field-of-view diffusion-weighted images with *b*-value 1500 s/mm^2^.

**Figure 3:**
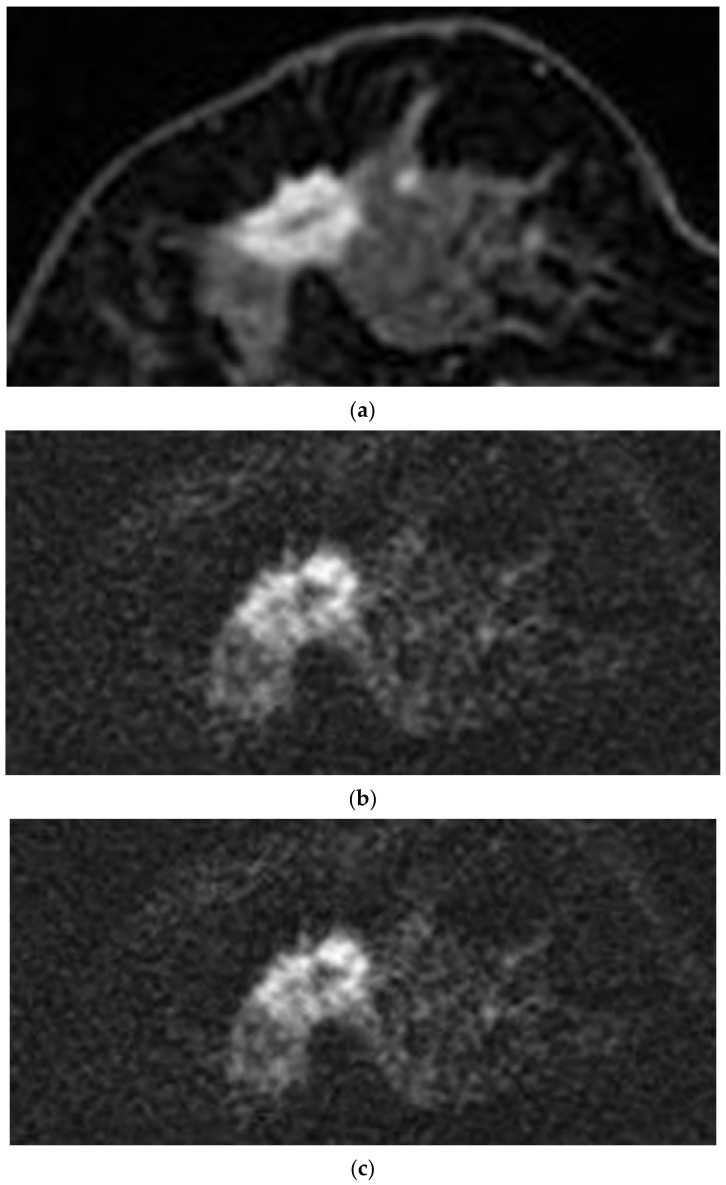
A 47-year old woman who was diagnosed invasive ductal carcinoma in her right breast. Axial contrast-enhanced T1-weighted fat-suppressed MRI (**a**) reveals a 16 × 12 mm-sized enhancing mass with irregular shape, spiculated margin, and rim, and marked enhancement is noted in the right breast at the 9 o’clock position. rFOVA-1000 ^a^ (**b**) and rFOVS-1000 ^b^ (**c**) also show index tumor well and had excellent depiction of shape irregularity and margin spiculation. ^a^ rFOVA-1000: acquired reduced field-of-view diffusion-weighted images with *b*-value 1000 s/mm^2^. ^b^ rFOVS-1000: synthetic reduced field-of-view diffusion-weighted images with *b*-value 1000 s/mm^2^.

**Figure 4:**
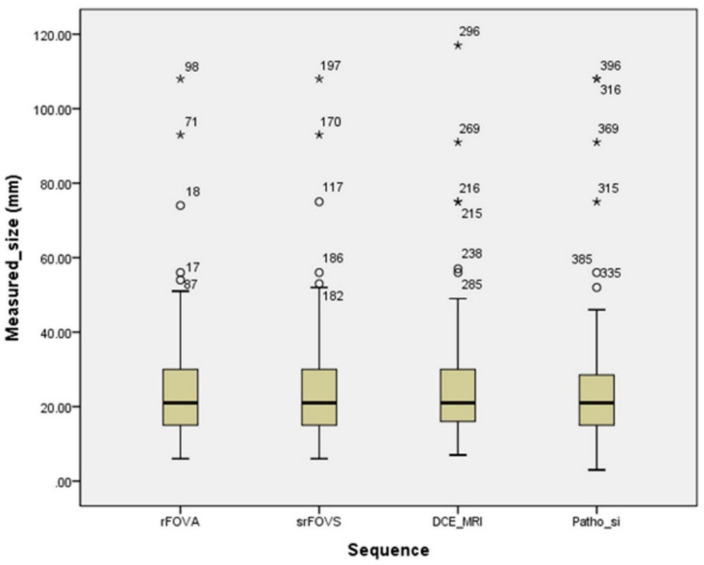
Boxplot shown the agreement among the size measurements of the index tumor on the srFOVS, rFOVA-1000, and DCE MRI and pathological tumor size. Rectangular box: interquartile range which contains the middle 50% of the measured size. Lines extend from the upper and lower edge of the box: whiskers which are no greater than 1.5 times of the interquatile range. Circle: out values greater than 1.5 times of the interquartile range. Stars: extreme values greater than 3 times of the interquartile range.

All 130 lesions were included in the qualitative analysis. Evaluation of the index tumor on rFOVA-1000 and srFOVS was difficult in only five cases. Evaluation on DCE MRI was also difficult in one of these five cases because of marked background parenchymal enhancement. The average score of rFOVA-1000 was 3.55; those of S-1000, S-1500, and S-2000 of rFOVS were 3.54, 3.07, and 2.08, respectively; and that of DCE MRI was 3.73. The image quality scores of rFOVA-1000 and DCE MRI were compared by selecting one sequence with the best image quality among the above-mentioned three sequences of rFOVS (srFOVS). The 130 cases of srFOVS were composed of 100 cases of S-1000, 30 cases of S-1500, and 0 of S-2000. Comparisons of the image quality scores on the basis of a four-point grading system among rFOVA-1000, rFOVS-1000, rFOVS-1500, rFVOS-2000, srFOVS, and DCE MRI are summarized in Table 2. The image quality scores of rFOVA-1000, srFOVS, and DCE MRI did not show significant difference (*p* = 0.357; Figure 2 and Figure 3). A total of 102 lesions were subjected to morphological analysis of the shape, margin, and size of the tumor. The other 28 lesions that were seen as non-mass enhancement on DCE MRI were excluded from the morphological analysis. The lesion interpretation of the index masses with regard to shape, margin, and size of the tumor also showed no statistically significant differences among rFOVA-1000, srFOVS, and DCE MRI (*p* = 0.858, *p* = 0.242, and *p* = 0.858, respectively) (Table 3, Figure 4).

### 3.3. Quantitative Analysis

A comparison of the quantitative parameters related to rFOVA-1000, srFOVS, and DCE MRI are shown in Table 4. SNR, CNR, and TPC of DCE MRI were significantly higher than that of rFOVA-1000 and srFOVS (*p* < 0.001, *p* = 0.001, and *p* = 0.016, respectively). However, there were no significant differences between rFOVA-1000 and srFOVS in terms of SNR, CNR, and TPC on post hoc analysis (*p* > 0.999, *p* > 0.999, and *p* > 0.999, respectively).

## 4. Discussion

In this study, we found that morphologic analysis and size measurement of the tumor could be performed well with rFOV DWI. In addition, with rFOVS, images equivalent to rFOVA could be obtained even with a relatively short image acquisition time. These results suggest that using rFOV DWI with computed DWI technique can provide efficient and accurate analysis of the breast tumor, even with the short image acquisition time.

Breast MRI has an important role in the preoperative setting for breast cancer patients or in the screening setting for high risk patients because of its higher cancer detection rate, which can be attributed to its excellent soft-tissue contrast compared with conventional modalities such as mammography and breast ultrasonography [37]. DBT, CEDM, CBBCT, and CEDBCT have been emerging techniques for breast imaging in recent times. DBT has improved mammographic sensitivity and specificity, however, the cancer detection rate of DBT was found to be significantly lower than breast MRI [38,39]. Moreover, DBT is vulnerable to axillary evaluation, which is essential in the preoperative evaluation. CEDM, CBBCT, and CEDBCT improved cancer diagnosis with high sensitivity and reduced false positive rate in recent studies [40,41,42,43,44,45,46,47,48,49]. Furthermore, virtual monoenergetic images acquired by using dual-layer spectral detector CT could be also useful in the diagnosis of breast cancers in patients who cannot have a breast MRI performed [50]. However, CEDM, CBBCT, and CEDBCT cannot avoid radiation exposure and the potential adverse reaction to CT contrast media. Moreover, the protocol of CEDBCT has not been estabilished yet, but the diagnostic accuracy may vary depending on the time of image acquisition, and thus it has been still somewhat insufficent to replace breast MRI in preoperative breast cancer patients.

DCE MRI requires the use of contrast agent; therefore, several studies have evaluated the diagnostic performance of unenhanced MRI, including DWI, finding it to be an acceptable alternative [12,14]. However, bilateral DWI has limitations, such as magnetic susceptibility and chemical shift artifacts, low SNR, and low resolution [17,51]. Therefore, various techniques have been suggested for minimizing these drawbacks. A few recent studies of breast imaging with rFOV DWI have shown that the images have to provide higher lesion conspicuity, better image quality, and relatively higher resolution compared to images obtained using conventional bilateral DWI [17,35,52]. On the basis of this point, we wanted to investigate if rFOV DWI could be used clinically instead of DCE MRI in breast cancer patients. Our study suggested that rFOV DWI did not differ significantly from DCE MRI in terms of morphological analysis of breast cancer.

In DWI, the images acquired with higher *b*-values show better image contrast due to increased suppression of the background signal, and therefore the lesion conspicuity of the malignancy is increased [23,53]. However, conventional DWI with high *b*-values have the disadvantages of low SNR, artifacts such as geometric distortion with large eddy currents, and relatively long image acquisition time [17,23,24] The recently introduced computed DWI technique, which improves the SNR and reduces artifacts, ameliorates the drawbacks of conventional DWI. There have been several studies regarding the clinical application of computed DWI in breast, liver, and prostate imaging [24,25,26,27,54,55]. Other recent studies have reported that computed DWI had the potential to improve the diagnostic sensitivity for breast cancer detection compared to acquired conventional DWI [24,25,26,27]. Our results show that the computed DWI images obtained from the ADC map calculated with acquired *b* = 0 s/mm^2^ and *b* = 500 s/mm^2^ DWI have similar image quality as that obtained with high *b*-value DWI in breast cancer patients. In addition, it provides the same level of information to the radiologist as DCE MRI with regard to morphological analysis of the breast tumor without injection of contrast media.

Moreover, the computed DWI technique enables a reduction of the image acquisition time by the simple technique of deriving a higher *b*-value DWI by extrapolating information obtained at two or more lower *b*-values from the calculated ADC map [22,23]. In this study, the image quality of the rFOV DWI with computed DWI technique was equivalent to that obtained with the acquired rFOV DWI with higher *b*-value, despite the short image acquisition time. The image acquition time using computed DWI technique was only reduced by 40 s. However, recently, the abbreviated breast MRI protocol has become important because of the possibility of a significant decrease in the breast MRI image acquisition time and cost of MRI [56]. From this point of view, it is clinically meaningful that a similar level of multiple image sets of various *b*-values could be obtained at once, even with a shorter acquisition time.

DWI, using *b*-values of 0 to 1000 s/mm^2^, has been widely used in the clinical setting [24]. Woodham et al. reported that the use of higher *b*-value DWI might aid in the visual analysis of the breast tumor [53]. In our study, among the S-1000, S-1500, and S-2000 of rFOVS, there was no case with higher S-2000 image quality score. However, in 30 patients, the image quality score of S-1500 was the highest among the image sets of rFOVS, as shown in Figure 2. The rFOVS enabled the images with the highest image quality to be selected and compared without additional image acquisition.

The results of our study support the idea that rFOV DWI with computed DWI technique could be the first step toward finding a potential alternative to DCE MRI. In the rFOV DWI with computed DWI technique, quantitative parameters such as SNR, CNR, and TPC were lower than those of DCE MRI, but were similar to those of the acquired higher *b*-value DWI. Unlike previous studies regarding computed DWI, in our study, the rFOV DWI with computed DWI technique did not show significant differences with regard to quantitative parameters and image quality scores compared to the acquired higher *b*-value rFOV DWI. The acquired higher *b*-value rFOV DWI is already known to have significantly higher image quality, higher lesion conspicuity, higher SNR, and less distortion than conventional DWI. Therefore, the scope for obtaining improved image quality and better quantitative parameters with the rFOV DWI with computed DWI technique would be limited. We believe that this is the main reason for the conflicting outcomes of the previous studies.

There were some limitations in our study. First, our study included only biopsy-confirmed maglinancies, which improved the diagnostic conspicuity. Thus, we could not evaluate the actual diagnostic capacity of rFOVA and rFOVS. However, the goal of this study was not to evaluate the actual diagnostic capacity of rFOVA and rFOVS, but to compare the inter-method agreement among rFOVA, rFOVS, and DCE MRI. Second, since rFOV DWI has a small field-of-view, it was not possible to evaluate bilateral and multiple cancer lesions on preoperative breast MRI. In order to solve this problem, it is neccessary to improve the image quality of T2-weighted images (T2WI) for evaluation of multiple lesions, and further studies on rFOV DWI and T2WI combination would be required. Third, we had to know the location of the target lesion and specify the field-of-view correctly. In some patients with large breast size or large target lesions, the lesions were not accurately included in the field-of-view. Therefore, re-imaging was necessary in those patients, and if they refused, they had to be excluded from the study. In other words, the radiologist had to confirm the localization image to designate and direct the position of the field-of-view to the radiologic technologist at the time of imaging acquisition. Fourth, several types of software have been used in the recent studies of computed DWI for breast cancer [24,25,26,27]. By conducting further studies using various software and various MRI systems, it would be necessary to validate our study and other previous studies about the computed DWI. Finally, qualitative analysis was performed by two radiologists in consensus, and quantitative analysis was performed by one radiologist. We did not evaluate the inter- and intraobserver variability for image quality scoring, morphological analysis, SNR, CNR, and TPC among rFOVA, rFOVS, and DCE MRI. Therefore, further studies by radiologists with various degress of experience in breast imaging are needed to evaluate intra- and interobserver variability.

In conclusion, both the rFOV DWI and rFOV DWI with computed DWI technique showed nearly equivalent levels of image quality required for morphological analysis of the tumors and for lesion conspicuity compared with DCE MRI. The rFOV DWI with computed DWI technique has the advantage of avoiding the use of contrast agents while reducing image acquisition time in breast MRI. The technique can have a useful clinical role in the morphological evaluation of the breast tumor and can be the first step in expanding the application of DWI as a potential alternative to DCE MRI.

## Figures and Tables

**Table 1:** Image acquisition parameters for reduced field-of-view (rFOV) diffusion-weighted imaging (DWI) and dynamic contrast-enhanced magnetic resonance imaging (DCE MRI).

Sequence Parameter	rFOV DWI		DCE MRI
	*b* = 500 s/mm^2^	*b* = 1000 s/mm^2^	
*b* value (s/mm^2^)	0, 500	0, 1000	Non-applicable
Fat suppression	Short tau inversion recovery	Short tau inversion recovery	Chemical shift-selective fat saturation
Repetition time (ms)	3000	3000	4.1
Echo time (ms)	52	58	1.7
Number of excitations	12	16	0.7
Acquisition matrix	128 × 64	128 × 64	320 × 320
Field-of-view (mm^2^)	100 × 50	100 × 50	320 × 320
Slice thickness (mm)	4	4	1
Intersection gap (%)	0	0	0
Acquisition time (s)	113	153	374
Flip angle (°)	90	90	10

**Table 2:** Comparisons of image quality score among acquired reduced field-of-view diffusion-weighted images *b*-value 1000 s/mm^2^ (rFOVA-1000), synthetic reduced field-of-view diffusion-weighted images, and dynamic contrast-enhanced magnetic resonance images (DCE MRI).

Image Quality	rFOVA-1000 ^a^	rFOVS-1000 ^b^	rFOVS-1500 ^c^	rFOVS-2000 ^d^	srFOVS ^e^	DCE MRI ^f^	*p*-Value
1 = poor	2	2	8	43	2	0	
2 = not good	6	6	29	41	6	1	
3 = minor degredation	41	41	39	39	29	32	
4 = no problem	81	81	54	7	93	97	
Average score	3.54 ± 0.66	3.54 ± 0.66	3.07 ± 0.94	2.08 ± 0.92	3.63 ± 0.65	3.73 ± 0.46	0.357

^a^ rFOVA-1000: acquired reduced field-of-view diffusion-weighted images with *b*-value 1000 s/mm^2^. ^b^ rFOVS-1000: synthetic reduced field-of-view diffusion-weighted images with *b*-value 1000 s/mm^2^. ^c^ rFOVS-1500: synthetic reduced field-of-view diffusion-weighted images with *b*-value 1500 s/mm^2^. ^d^ rFOVS-2000: synthetic reduced field-of-view diffusion-weighted images with *b*-value 2000 s/mm^2^. ^e^ srFOVS: synthetic reduced field-of-view diffusion-weighted images with selected *b*-value. ^f^ DCE MRI: dynamic contrast-enhanced magnetic resonance images.

**Table 3:** Comparisons of morphological analysis and size measurement of index cancer among rFOVA-1000, srFOVS, and DCE MRI.

Lesion Analysis	rFOVA-1000 ^a^	srFOVS ^b^	DCE MRI ^c^	*p-*Value
Shape				0.940
Oval	14	12	11	
Round	7	8	8	
Irregular	79	80	81	
Margin				0.662
Circumscribed	9	9	17	
Irregular	55	56	49	
Spiculated	38	37	36	
Non-mass enhancement	28	28	28	
Average tumor size (mm)	25.5 ± 16.15	25.7 ± 16.17	26.3 ± 17.34	0.585

^a^ rFOVA-1000: acquired reduced field-of-view diffusion-weighted images with *b*-value 1000 s/mm^2^.^b^ srFOVS: synthetic reduced field-of-view diffusion-weighted images with selected *b*-value. ^c^ DCE MRI: dynamic contrast-enhanced magnetic resonance images.

**Table 4:** Comparisons of quantitative parameters among rFOVA-1000, srFOVS, and DCE MRI.

	rFOVA-1000 ^a^	srFOVS ^b^	DCE MRI ^c^	*p*-Value
Signal-to-noise ratio (SNR)	16.21 ± 5.82	16.06 ± 5.88	85.03 ± 20.97	<0.001
Contrast-to-noise ratio (CNR)	2.02 ± 1.00	1.98 ± 0.98	2.53 ± 1.01	0.001
Tumor-to-parenchymal contrast (TPC)	2.33 ± 0.87	2.35 ± 0.94	2.79 ± 1.66	0.016

^a^ rFOVA-1000: acquired reduced field-of-view diffusion-weighted images with *b*-value 1000 s/mm^2^. ^b^ srFOVS: synthetic reduced field-of-view diffusion-weighted images with selected *b*-value. ^c^ DCE MRI: dynamic contrast-enhanced magnetic resonance image.
